# The risk of rheumatoid arthritis among patients with inflammatory bowel disease: a systematic review and meta-analysis

**DOI:** 10.1186/s12876-020-01339-3

**Published:** 2020-06-17

**Authors:** Yi Chen, Lang Chen, Changsheng Xing, Guangtong Deng, Furong Zeng, Tingting Xie, Lei Gu, Huixiang Yang

**Affiliations:** 1 Department of Gastroenterology, Xiangya Hospital, Central South University, 87 Xiangya Road, Changsha, 410008 Hunan Province China; 2 Department of General Surgery, The Third Xiangya Hospital, Central South University, 138 Tongzipo Road, Changsha, 410013 Hunan China; 3 Center for Inflammation and Epigenetics, Houston Methodist Research Institute, 6670 Bertner Avenue, Houston, TX 77030 USA; 4 Department of General Surgery, Xiangya Hospital, Central South University, 87 Xiangya Road, Changsha, 410008 Hunan China; 5 Department of Rheumatology, Xiangya Hospital, Central South University, 87 Xiangya Road, Changsha, 410008 Hunan China

**Keywords:** Crohn disease, Inflammatory bowel disease, Meta-analysis, Rheumatoid arthritis, Ulcerative colitis

## Abstract

**Background:**

Studies have suggested that patients with inflammatory bowel disease (IBD) have an increased risk of rheumatoid arthritis (RA). However, the available data on this association are inconsistent. This meta-analysis aimed to determine the association between IBD and the risk of RA.

**Methods:**

Observational studies investigating the RA risk among patients with IBD (Crohn disease (CD) and/or ulcerative colitis (UC)) were searched in PubMed, Embase, and Web of Science from the date of inception to December 2019. The methodological quality of the included studies was assessed using the Newcastle-Ottawa Scale. Relative risks (RRs) and corresponding 95% confidential intervals (CIs) were pooled with a random-effects model. Heterogeneity was evaluated using I^2^ statistics while publication bias was determined using Begg’s and Egger’s tests. Subgroup and sensitivity analyses were performed.

**Results:**

A total of three cohort studies, three cross-sectional studies, and two case-control studies were included in the meta-analyses. Compared to the non-IBD control or general population, there was a significantly higher risk of RA among patients with IBD (RR = 2.59; 95% CI: 1.93–3.48). Moreover, both CD (RR = 3.14; 95% CI: 2.46–4.01) and UC (RR = 2.29; 95% CI: 1.76–2.97) were associated with a significantly increased risk of RA. However, heterogeneity was substantial across studies and the subgroup analyses failed to identify the potential source of heterogeneity.

**Conclusions:**

Patients with IBD have a greater risk of developing RA. Rheumatologists should be consulted when patients with IBD present with undifferentiated joint complaints. However, more prospective cohort studies are needed to validate these results.

## Background

Inflammatory bowel disease (IBD), which comprises Crohn disease (CD) and ulcerative colitis (UC), is a chronic non-specific inflammatory condition of the gastrointestinal tract (GI) [1]. While CD is characterized by discontinuous and transmural inflammation, which can affect all parts of the gastrointestinal tract, UC causes a continuous and superficial inflammation of the large intestine [1]. Besides the pathological changes and discomfort in the GI tract, up to 50% of the patients with IBD experience extra-GI manifestations, the most common being arthritis [2–4]. The majority of IBD-related arthritis cases parallel IBD activity and are self-limited and non-erosive [5].

Rheumatoid arthritis (RA) is a chronic synovitis-based systemic disease of unknown etiology, which is characterized by symmetrical invasive inflammation of multiple joints of the body [6]. Without early and adequate treatment, deformity and dysfunction of the affected joints due to severe bone destruction and absorption will occur in the late stage [7]. However, the signs and symptoms of RA may be quite similar to those of IBD-related arthritis, especially at the early stage of RA. Therefore, it may be difficult to distinguish them in the clinical setting, which may delay the diagnosis and treatment of RA. Moreover, as both IBD and RA are progressive and disabling diseases, the co-occurrence of these two conditions, therefore, severely increases the disease burden and compromises the quality of life and prognosis of these patients [8].

In recent years, an increasing number of studies have noted that RA tended to cluster with IBD [9–14] and that patients with IBD are more likely to develop RA [15–18]. In contrast, a nationwide population-based case-control study indicated there was a decreased risk of RA in patients with IBD, although the decrease was not statistically significant [19]. Moreover, there is controversy regarding the risk of RA in patients with IBD when the association between RA and CD and between RA and UC is analyzed separately [11]. However, a systematic evaluation and quantitative synthesis of studies on the association between IBD and RA is still not available. Evaluating the current evidence on the association between them will facilitate our understanding of both conditions and provide a reference for better healthcare practices. Therefore, we aimed to conduct a meta-analysis to systematically analyze the association between IBD and the risk of RA; we discussed the underlying mechanisms as well. To the best of our knowledge, this is the first systematic review and meta-analysis focusing on the association between IBD with RA.

## Methods

This study was performed according to the Preferred Reporting Items for Systematic Reviews and Meta-Analyses (PRISMA) [20] and Meta-analysis of Observational Studies in Epidemiology (MOOSE) guidelines [21].

### Search strategy

Related publications were comprehensively searched in online databases (including PubMed, Embase, and Web of Science) from the inception date to December 2019 by two independent reviewers (YC and LC). Terms used in the search included a combination of the following terms and their corresponding synonyms: “inflammatory bowel disease”, “Crohn disease”, “ulcerative colitis”, and “rheumatoid arthritis”. Moreover, the references of relevant reviews and included studies were screened manually to prevent omission. The detailed search strategy is shown in supplementary Table 1 (Additional file 1).

### Study selection

We included studies meeting the following inclusion criteria: 1) written in English; 2) cross-sectional, case-control, or cohort studies which reported the risk estimates of RA among patients with IBD (CD and/or UC), or presented data to calculate these effect sizes; 3) cross-sectional or cohort studies containing a control group consisting of individuals without IBD or the general population; case control studies containing a control group consisting of individuals without RA or the general population. If different publications utilized duplicated data, the most comprehensive and newest one was included in this study. If different publications recruited overlapping population from the same database, we included the one with the best methodological quality in the statistical synthesis. Cohort study was considered as the best quality design, followed by case-control and cross-sectional study. The quality of studies with the same design was ranked by the quality assessment scale mentioned below. We excluded any reviews, expert opinions, case reports, and case series.

All the retrieved publications were exported to EndNote for selection. After removal of duplicated studies, the titles and abstracts of unique publications were screened to discard ineligible studies. Next, the full text of the remaining articles was checked for their eligibility according to the selection criteria. The entire selection process was independently performed by two authors (YC and LC) and any disagreement was resolved by discussion and a third reviewer (GD) was consulted when necessary.

### Data extraction

The following information was extracted from eligible studies: last name of the first author, year of publication, country of the population studied, study design, study duration, source of case and control groups, definition criteria for IBD and RA, age and gender of the population, sample size of each group, and effect sizes of the association between IBD and RA. Data were independently extracted and cross-checked by two investigators (YC and LC).

### Quality assessment

The methodological quality of included case-control and cohort studies on the association between IBD and the risk of RA was assessed using the Newcastle-Ottawa Scale [22], which included 3 domains: selection (4 items, 1 point for each item), comparability (1 item with 2 points), and ascertainment of exposure or outcome (3 items, 1 point for each item). A modification of the Newcastle-Ottawa Scale as described by Herzog et al. [23] was used for the assessment of cross-sectional studies. Studies that scored ≥6 points were considered to be of good quality. The quality assessment was performed independently by 2 investigators (YC and LC), with disagreements resolved by discussion or consulting another investigator (GD).

### Statistical analysis

Stata software version 12.0 (StataCorp, College Station, TX) was employed to conduct all the meta-analyses. Odds ratio, hazards ratio, and incident rate ratio were assumed to be similar estimates of relative risk (RR) given that both IBD and RA are relatively rare diseases [24]. If more than one risk estimate was presented in a study, the most adjusted one was used for the meta-analysis. All summary estimates were calculated with a DerSimonian-Laird random-effects model [25] due to the anticipated high level of study heterogeneity. Heterogeneity among studies was assessed by means of the I^2^ statistics, in which I^2^ > 50% was regarded as a marker of statistically significant heterogeneity. Additionally, subgroup analyses were conducted to better assess between-study variability and explore the potential impact factors. Sensitivity analysis was performed by excluding one study at a time and pooling the prevalence and risk estimates for the remaining studies, to evaluate the stability of the summary estimates. Publication bias was assessed with Egger’s test [26] and Begg’s test [27]. *P* < 0.05 was considered statistically significant in all cases, except for the publication bias test, in which the threshold value was set at 0.10.

## Results

### Study selection

**Fig. 1:**
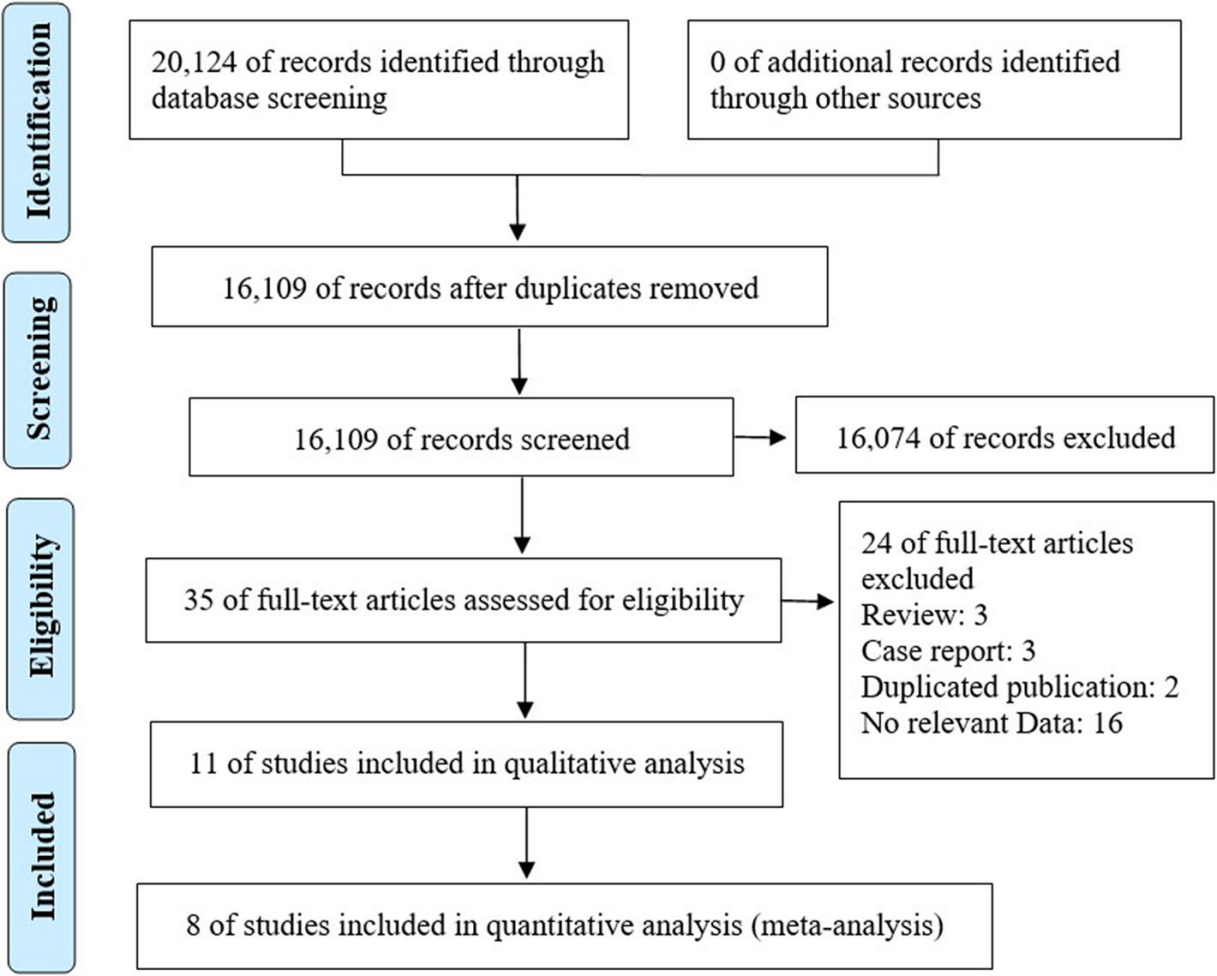
Flowchart of the literature selection process

The electronic literature search identified 20,124 publications. The titles and abstracts of 16,109 unique studies were screened using the selection criteria, leaving 35 papers. The full texts of these articles were carefully reviewed. Finally, 11 studies meeting the selection criteria were selected for further qualitative analysis, as shown in Fig. 1.

### Study characteristics

The characteristics of these studies are outlined in Tables 1 and 2. The studies were published between 2007 and 2019 and conducted in the USA [10, 11, 13, 17, 18], Korea [9, 14, 15], Denmark [12, 16], and Finland [19]. Three publications [15–17] were retrospective cohort studies, two [18, 19] were case-control studies and the other six [9–14] were cross-sectional studies. Six studies [9, 12, 14–16] were based on nationwide population-based databases, four [10, 11, 13, 17] were based on large administrative databases and the other one [18] was based on single-center biobank. Two cross-sectional studies [9, 14] and one cohort study [15] recruited an overlapping population from the Korean National Health Insurance Database; we only included the cohort study in the statistical analysis. Likewise, since two studies [12, 16] recruited overlapping samples from the Danish National Patient Database, we only included the cohort study in the meta-analysis. The cross-sectional study conducted by Cohen et al. [13] utilized two independent datasets which were marked as Cohen-2008-1 and Cohen-2008-2, respectively. Dataset Cohen-2008-1 was only included in the meta-analysis of risk of RA in patients with CD or UC alone, as it shared an overlapping sample with the cohort study conducted by Aletaha et al. [17] when investigated the risk of RA in patients with IBD.

**Table 1:** Characteristics of the included studies-Part I

Study	Country	Design/study duration	Sample source	Definition criteria	
				IBD	RA
Aletaha-2019 [17]	USA	Retrospective Cohort/10 years	MarketScan Commercial Claims and Encounters database	≥2 claims at least 30 days apart with ICD-9 codes (555.x, 556.x)	≥2 claims at least 30 days apart with ICD-9 codes (714, 714.0, 714.8, 714.89, 714.9)
Bae-2017^a^ [14]	Korea	Cross-sectional/NA	National Health Insurance Database	≥5 health contacts due to UC/ CD as the principal diagnosis with ICD-9 codes (K50, K51)	≥5 health contacts due to RA as the principal diagnosis with ICD-9 codes (M05, M06)
Burisch-2019 [16]	Denmark	Retrospective Cohort/10 years	National Patient Registry	≥2 diagnoses with ICD-8 codes (563.01, 563.19) or ICD-10 codes (K50, K51)	≥1 diagnosis with ICD-10 codes (M05, M06.0, M06.8, M06.9)
Cohen-2008-1 [16]	USA	Cross-Sectional/ NA	IMS Health Integrated Administration Claims Database	≥1 claim with ICD-9 codes (555.x, 556.x)	≥1 claim with ICD-9 codes (714.x)
Cohen-2008-2 [13]	USA	Cross-Sectional/ NA	MarketScan Commercial Claims and Encounters Database	≥1 claim with ICD-9 codes (555.x, 556.x)	≥1 claim with ICD-9 codes (714.x)
Halling-2017^a^ [12]	Denmark	Cross-Sectional/ NA	National Patient Registry	ICD-10 codes (K50, K51)	ICD-10 codes
Kappelman-2011 [11]	USA	Cross-Sectional/ NA	PharMetrics Patient-Centric Database	≥3 health contacts with associated ICD-9 codes (555.x, 556.x), or ≥ 1 claim with associated medication history	ICD-9 codes
Park-2019 [15]	Korea	Retrospective cohort/4 years	National Health Insurance Database	ICD-10 codes (K50, K51) and registration codes (V130, V131) for the rare/intractable disease patient-support program	ICD-10 codes and registration codes for the rare/intractable disease patient-support program
Puolakka-2014 [19]	Finland	Case-control/4 years	National Registry of the Social Insurance Institution	ICD-10 codes (K50, K51)	ICD-10 codes (M05, M06)
Kronzer-2019 [18]	USA	Case-control/10 years	Single-center biobank	Questionnaire	≥2 diagnoses at least 30 days apart with ICD-9 codes (714.0, 714.9) plus use of a disease modifying antirheumatic drug
Weng-2007 [10]	USA	Cross-sectional/NA	Northern California region of the Kaiser Permanente Medical Care Plan	≥2 diagnoses with ICD-9 codes (555.x, 556.x)	≥2 diagnoses with ICD-9 codes (714.0, 714.3x)
Yang-2018^a^ [9]	Korea	Cross-Sectional/NA	National Health Insurance Database	ICD-10 codes (K50, K51) and registration codes (V130, V131) for the rare/intractable disease patient-support program	ICD-10 codes (M05, M06)

*CD* Crohn Disease, *IBD* Inflammatory Bowel Diseases, *NM* Not Mentioned, *ICD-8* International Classification of Disease 8th revision, *ICD-9* International Classification of Disease 9th revision, *ICD-10* International Classification of Disease 10th revision, *NA* Not Apply, *RA* Rheumatoid Arthritis, *UC* Ulcerative Colitis

^a^These cross-sectional studies were not included in the statistical meta-analysis as the populations in these studies overlapped with those in the other cohort studies

**Table 2:** Characteristics of the included studies-Part II

Study	Case type	Sample (size (n)/Age range (years)/Male (%))		Adjusted RR (95%CI)	Covariates adjustment	NOS score
		Cases	Controls			
Aletaha-2019 [17]	IBD	68,535/18–64/NM	42,371,769/18–64/NM	3.5 (3.3–3.8)	Age, sex, region, and insurance	7
Bae-2017 [14]	IBD	40,843/All/60.2	122,529/All/60.2	3.41 (2.61–4.44)	Age, sex, and insurance	7
	CD	12,646/All/69.1		3.74 (2.47–5.65)		
	UC	28,197/All/59.2		3.31 (2.49–4.40)		
Burisch-2019 [16]	IBD	11,777/≥18/NM	1 for 5 matched^a^ /≥18/NM	2.11 (1.66–2.67)	Age, sex	8
	CD	NM/≥18/NM		3.59 (2.32–5.54)		
	UC	NM/≥18/NM		1.67 (1.21–2.30)		
Cohen-2008-1 [13]	IBD	18,603/≥18/44.3	66,969/≥18/44.3	2.72 (2.43–3.04)	Age, sex, region, and insurance	7
	CD	9267/≥18/43.0	37,068/≥18/43.0	2.76 (2.40–3.18)		
	UC	11,220/≥18/46.0	44,880/≥18/46.0	2.76 (2.40–3.18)		
Cohen-2008-2 [13]	IBD	16,139/≥18/45.5	64,556/≥18/45.5	2.05 (1.84–2.29)	Age, sex, region, and insurance	7
	CD	7404/≥18/42.7	29,604/≥18/42.7	2.37 (2.02–2.77)		
	UC	10,104/≥18/45.7	40,416/≥18/45.7	2.16 (1.88–2.49)		
Halling-2017 [12]	IBD	47,325/All/46	92,839/All/46	1.8 (1.5–2.0)	Age, sex, and region	8
	CD	13,343/All/42	26,172/All/42	2.1 (1.6–2.8)		
	UC	31,066/All/47	60,951/All/47	1.6 (1.3–1.9)		
Kappelman-2011 [11]	IBD	1242/≤20/55	3353/≤20/54	9.6 (3.9–23.8)	Age, sex, and region	8
	CD	737/≤20/56	1997/≤20/56	15.7 (4.6–53.7)		
	UC	488/≤20/53	1310/≤20/52	3.6 (0.8–16.1)		
Park-2019 [15]	IBD	35,581/≤80/61.6	142,324/≤80/61.6	4.24 (3.25–5.52)	Age, sex	8
	CD	11,803/≤80/70.9	47,212/≤80/70.9	5.59 (3.32–9.41)		
	UC	23,737/≤80/56.9	94,948/≤80/56.9	3.90 (2.86–5.32)		
Puolakka-2014 [19]	RA	7209/≥17/32	NM/NM/NM	0.74 (0.51–1.03)	Age, sex	7
Kronzer-2019 [18]	RA	821/≥18/27	2455/≥18/27	3.82 (1.77–8.41)	Age, sex, region, race, BMI, education, smoking, CCI	6
Weng-2007 [10]	IBD	12,601/≤89/47.5	50,404/≤89/47.5	1.9 (1.5–2.3)	Age, sex, insurance, and smoking	9
	CD	4021/≤89/44.1	1 for 4 matched/≤89/44.1	2.5 (1.8–3.5)		
	UC	7525/≤89/49.3	1 for 4 matched/≤89/49.3	1.5 (1.2–2.0)		
Yang-2018 [9]	IBD	43,281/19–75/70.1	1,127,261/19–75/48.4	1.60 (1.52–1.68)	No	6
	CD	13,925/19–75/63.5		1.47 (1.34–1.61)		
	UC	29,356/19–75/57.6		1.67 (1.57–1.77)		

*BMI* Body Mass Index, *CCI* Charlson Comorbidity Index, *CD* Crohn Disease, *IBD* Inflammatory Bowel Diseases, *RA* Rheumatoid Arthritis, *RR* Relative Risk, *UC* Ulcerative Colitis

^a^The sample size of CD and UC was not mentioned; however, each patient was paired with 5 age- and sex-matched 5 individuals from the general population

The final datasets for evaluating the risk of RA among IBD consisted of 42,987,815 participants (193,200 nonoverlapping IBD patients). Besides, a cumulative total of 204,712 participants (46,575 nonoverlapping CD patients) and 356,745 participants (84,140 nonoverlapping UC patients) were included in the meta-analysis on the association between CD and RA, and between UC and RA, respectively. Regarding gender distribution, the proportion of male ranged from 27 to 70%. However, the gender information of 2 studies [16, 17], including the one [17] with the biggest sample size, was not reported. One study [11] focused on the children, five [13, 16–19] analyzed adult population, and the remaining studies [12, 15] recruited both children and adults.

### Study quality

A summary of the methodological quality scores of the included studies is shown in Table 2 and the detailed information is presented in supplementary Table 2 (Additional file 2). With respect to the risk of RA among patients with IBD, 10 studies with 11 datasets showed “good quality”, with a median score of 7 (range: 6–9).

### Risk of RA in patients with IBD

**Fig. 2:**
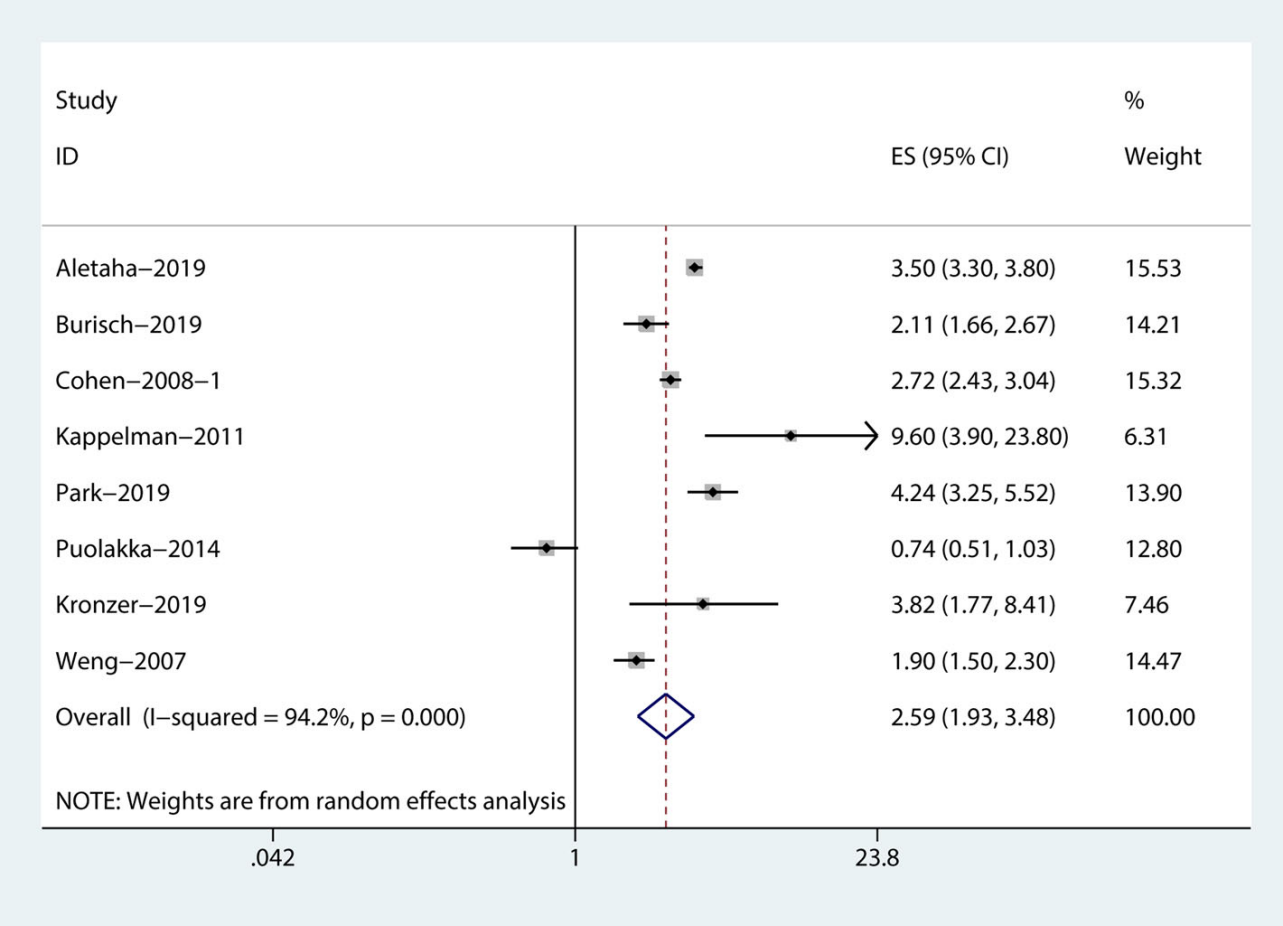
Forest plots on the risk of rheumatoid arthritis among patients with inflammatory bowel disease

**Fig. 3:**
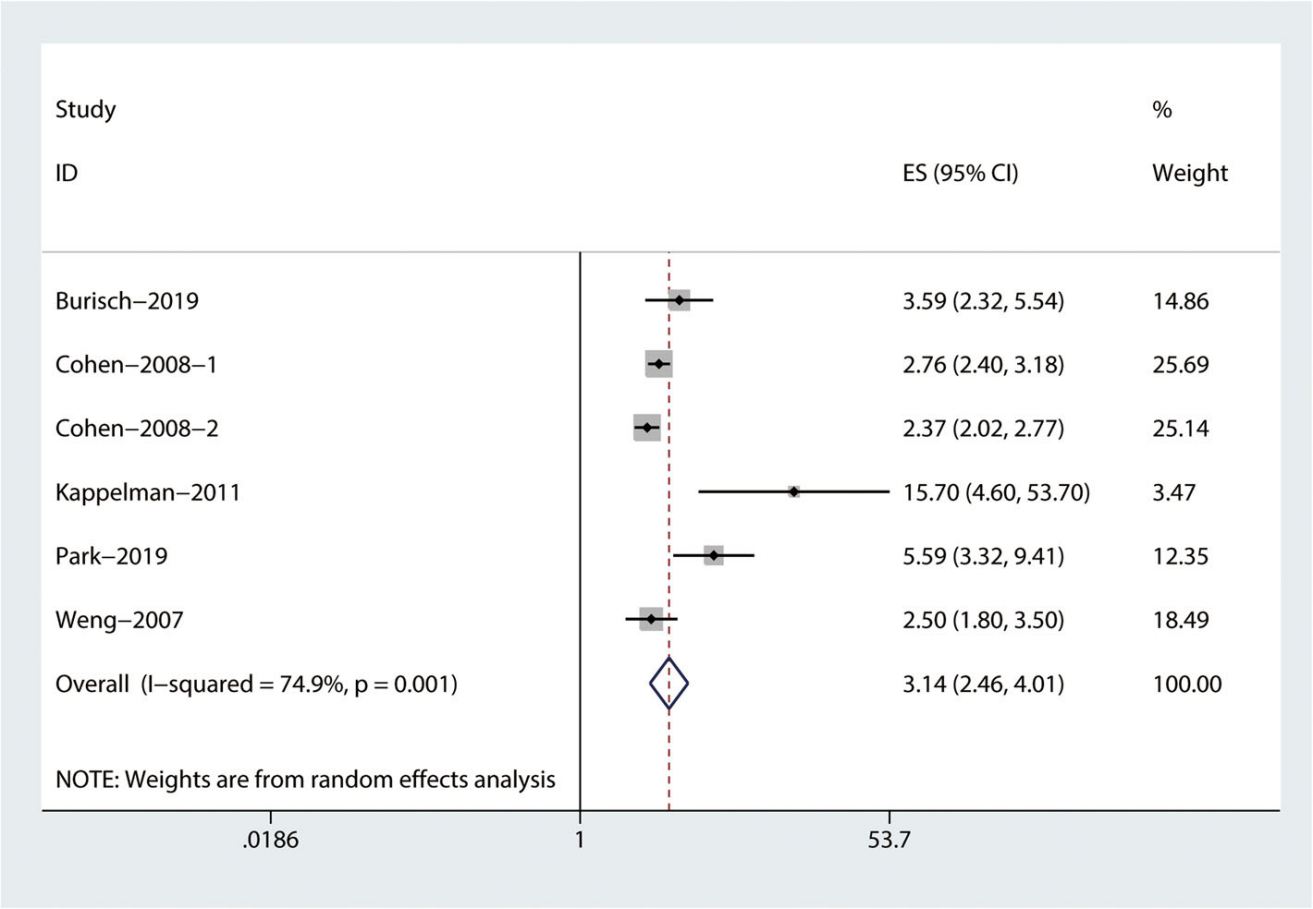
Forest plots on the risk of rheumatoid arthritis among patients with Crohn disease

**Fig. 4:**
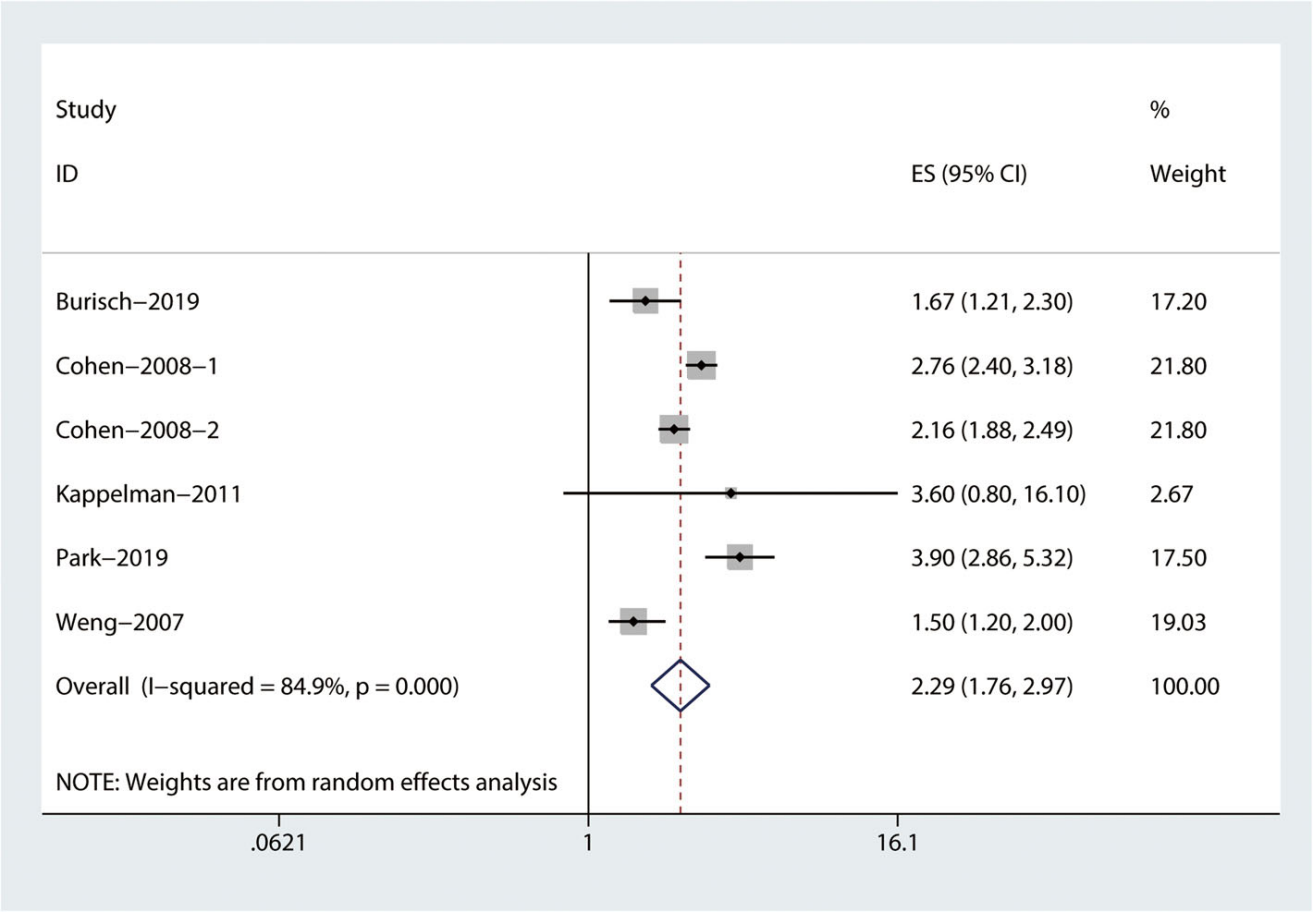
Forest plots on the risk of rheumatoid arthritis among patients with ulcerative colitis

The combined evidence from eight studies (three cohort studies [15–17], two case-control studies [18, 19], and three cross-sectional studies [10–12]) showed a significant increased risk of RA among patients with IBD (RR = 2.59, 95% CI: 1.93–3.48, I^2^ = 94.2%; Fig. 2). In addition, the pooled risk estimates of six datasets from five studies showed that the corresponding risks were both significantly increased in patients with CD (RR = 3.14, 95% CI: 2.46–4.01, I^2^ = 74.9%; Fig. 3), and UC (RR = 2.29, 95% CI: 1.76–2.97, I^2^ = 84.7%; Fig. 4).

Subgroup analyses stratified by study design, age of the targeted population, year of publication, and study duration were performed. Results showed that the risk of RA among patients with IBD was significantly increased in all subgroups except for the studies with case-control design (RR = 1.62, 95% CI: 0.32–8.07, I^2^ = 92.9%; Table 3) and those with shorter study duration (RR = 1.76, 95% CI: 0.31–9.92, I^2^ = 98.4%). The corresponding risk in CD remained significantly increased in all subgroups. The association between UC and the risk of RA was less significant in the pediatric group (RR = 3.60, 95% CI: 0.80–16.10) and in the group comprised of both children and adults (RR = 2.41, 95% CI: 0.94–6.14, I^2^ = 95.4%). However, we failed to identify the source of heterogeneity based on these factors.

**Table 3:** Subgroup analysis on the risk of RA in IBD

Subgroup analysis	No. of Studies	Pooled estimates (random-effects model)		
		Relative risk (95% CI)	***P*** value	I^**2**^ statistics
**The risk of RA in IBD**	8	2.59 (1.93–3.48)	0.000	94.2%
Study design				
Cross-sectional	3	2.84 (1.85–4.37)	0.000	88.1%
Case-control	2	1.62 (0.32–8.07)	0.557	92.9%
Retrospective cohort	3	3.16 (2.26–4.41)	0.000	89.4%
Age				
Children	1	9.60 (3.90–23.80)	0.000	–
Adult	5	2.20 (1.53–3.17)	0.000	95.6%
Children and adult	2	2.83 (1.29–6.21)	0.010	95.3%
Publication year				
Recent 5 years	5	2.42 (1.45–4.02)	0.001	95.5%
Over 5 years	3	2.84 (1.85–4.37)	0.000	88.1%
Study duration				
10 years	3	2.94 (1.91–4.52)	0.000	88.3%
4 years	2	1.76 (0.31–9.92)	0.522	98.4%
**The risk of RA in CD**	6	3.14 (2.46–4.01)	0.000	74.9%
Study design				
Cross-sectional	4	2.71 (2.14–3.42)	0.000	70.9%
Retrospective cohort	2	4.37 (2.84–6.73)	0.000	38.8%
Age				
Children	1	15.70 (4.60–53.70)	0.000	–
Adult	3	9.59 (3.86–23.83)	0.000	51.0%
Children and adult	2	3.64 (1.66–8.00)	0.001	84.6%
Publication year				
Recent 5 years	2	4.37 (2.84–6.73)	0.000	38.8%
Over 5 years	4	3.17 (2.05–4.90)	0.000	75.1%
**The risk of RA in UC**	5	2.29 (1.76–2.97)	0.000	84.7%
Study design				
Cross-sectional	4	2.15 (1.62–2.86)	0.000	83.6%
Retrospective cohort	2	2.55 (1.11–5.87)	0.000	92.8%
Age				
Children	1	3.60 (0.80–16.10)	0.094	–
Adult	3	2.22 (1.74–2.84)	0.000	81.4%
Children and adult	2	2.41 (0.94–6.14)	0.066	95.4%
Publication year				
Recent 5 years	2	2.55 (1.11–5.87)	0.027	92.8%
Over 5 years	4	2.15 (1.62–2.86)	0.000	83.6%

*CD* Crohn Disease, *IBD* Inflammatory Bowel Disease, *RA* Rheumatoid Arthritis, *UC* Ulcerative Colitis

### Publication bias and sensitivity analysis

No significant publication bias was found in the meta-analysis on the risk of RA among patients with IBD or UC. However, significant publication bias was detected in the meta-analysis on the association between CD and the risk of RA (Begg’s test: *P* = 0.060; Egger’s test: *P* = 0.048; Supplementary Fig. 1, Additional file 3).

The results of sensitivity analysis showed that the risk estimates of RA among patients with IBD, and with CD or UC alone, were stable (Supplementary Table 3, Additional file 4).

## Discussion

Previous studies have proposed a link between IBD and RA. However, whether there is an increased risk of RA among patients with IBD remains controversial. In this study, we systematically reviewed the published literature and analyzed the association between IBD and the risk of RA. Our results showed that there was a significantly increased risk of RA in patients with IBD (RR = 2.59, 95% CI: 1.93–3.48). Moreover, the increase in risk remained significant when CD and UC were separately analyzed (CD: RR = 3.14, 95% CI 2.46–4.01; UC: RR = 2.29, 95% CI 1.76–2.97).

Although there was a similar trend in the correlation between IBD and RA among almost all the datasets included in this study, the effect sizes varied greatly among studies and considerable heterogeneity was detected. We performed subgroup analyses based on the study design, age of the recruited anticipants, year of publication, and study duration, but failed to identify the source of heterogeneity. The heterogeneity may be partly explained by the different studied populations, disease definition criteria, and covariate adjustment. However, these factors could not be effectively stratified due to limited datasets. To generate more conservative results, the random-effects model was applied in all scenarios. In cross-sectional studies, the IBD and RA were measured during the same timeframe and therefore provide weaker evidence than longitudinal studies for the causal relationship between IBD and RA. However, this issue did not affect the findings, since in the subgroup analysis stratified by study design, the correlations between IBD and the increased risk of RA were consistently significant in both cross-sectional and cohort studies.

The uncovering of shared susceptibility loci and DNA polymorphisms has explained the relationship between IBD and RA at a genetic level. It has been reported that polymorphisms in the IRF5 locus can confer susceptibility to UC, CD, and RA [28–30]. Meanwhile, studies have determined that IL2/IL21 and TNFRSF14 loci are common risk loci for RA and UC [31–34]. Likewise, the risk loci shared by CD and RA have been identified in two genome-wide association studies [35, 36]. Proteins encoded by these shared risk alleles are key modulators of immune and inflammation, mainly involving the activation and differentiation of T cell (e.g., IL2 and IL21) [31], and pro-inflammatory signaling (e.g., IRF5 and TNFRSF14) [37, 38].

It has been suggested that a chronically imbalanced mucosal immune response to gut microbiota in the genetically susceptible patients may play an important role in the pathogenesis of IBD [39–42]. A few studies indicated that there was a link between gut microbiota and RA as well. Bacteroides is one of the dominant symbiont bacteria in the human intestine, which can induce regulatory T cell and cytokines that are protective against colitis [43]. It has been reported that Bacteroides was decreased in amount in both IBD and RA patients [44]. Additionally, microbiota-dependent T helper type 17 cells (Th17 cells) induction have been implicated in both conditions [45, 46]. Studies utilizing animal models demonstrated that RA could be rescued by eliminating the bacteria residing in the intestine, while treating sterile mice with a specific intestine-residing bacterium, segmented filamentous bacteria, led to the development of RA via Th17 cells [46].

CD and UC are conventionally considered to be a Th1-dominant condition and a Th2-dominant condition, respectively [47, 48]. Whereas RA is conventionally designated as a Th1-driven disease [49]. However, succedent evidences revealed that the immunological mechanisms of these inflammatory diseases seemed to be much more complex, with an emphasis on the interplay between dendritic cells and Th17 cells [50–53]. In addition, IL-23/IL-17 axis is regard as the pivotal dysregulated signaling pathway across CD, UC and RA [54–56].

RA is a chronic and progressive disease; early diagnosis and intervention are essential for better prognosis. The management strategy of RA has focused on the early identification of high-risk patients [57, 58]. Rheumatoid arthritis is currently diagnosed mainly according to the 2010 European League Against Rheumatism (EULAR)/American College of Rheumatology (ACR) RA classification criteria [59], which comprise four modules, namely joint involvement, duration of symptoms, serology and acute phase reactants. In general, joint symptoms and signs including morning stiffness lasting over half an hour, swelling, and objective evidence of synovitis would favor a diagnosis of RA [59]. However, these typical symptoms and signs may be lacking and RA-specific marker such as rheumatoid factor and anti-citrullinated protein antibody may be negative in the patients with RA especially during the early phase [60, 61]. It might be difficult for clinicians to distinguish arthritis related to IBD and early RA due to the obscure signs and symptoms. Therefore, collaboration between gastroenterologists and rheumatologists, and close follow-up and reassessment are suggested for the management of IBD patients presenting with undifferentiated arthritis.

The results presented in this meta-analysis must be interpreted with caution due to the several limitations of the study. First, our results were based on cross-sectional, case-control, and retrospective cohort studies; no prospective cohort study was available. Second, only a few studies were included in our meta-analyses. However, this could be partly compensated for by the large number of patients as all the studies included were based on nationwide or large-scale databases. Third, while smoking is the most important environmental risk factor for RA, only 2 studies [10, 18] included in this meta-analysis adjusted for the smoking status between the IBD group and the non-IBD control group. Fourth, there was considerable heterogeneity in our meta-analysis and we failed to identify the source of heterogeneity in the subgroup and sensitivity analyses. Fifth, significant publication bias was observed in the meta-analysis of the association between CD and risk of RA. Sixth, only the studies from USA, Denmark, Finland, and Korea analyzed the risk of RA in patients with IBD, which may compromise the generalizability of the results for the populations of other countries.

## Conclusion

Our study results indicate there is a significant association between IBD and the risk of RA. The findings of this meta-analysis suggest that rheumatologist consultation may be indicated when patients with IBD present with undifferentiated joint complaints. However, more prospective cohort studies are needed to validate these results.

## Supplementary information

**Additional file 1 Supplementary Table 1.** Search strategies.

**Additional file 2 Supplementary Table 2.** Quality assessment the of included studies.

**Additional file 3 Supplementary Figure 1.** Publication bias assessed by Begg’s test and Egger’s test.

**Additional file 4 Supplementary Table 3.** Sensitivity analysis.

## Data Availability

The datasets supporting the conclusions of this article are included within the article and its additional files.

## Abbreviations

ACR: American College of Rheumatology
CD: Crohn Disease
CI: Confidence Interval
EULAR: European League Against Rheumatism
GI: Gastrointestinal Tract
IBD: Inflammatory Bowel Disease
I^2^: I-squared
MOOSE: Meta-analysis of Observational Studies in Epidemiology
PRISMA: Preferred Reporting Items for Systematic Reviews and Meta-Analyses
RA: Rheumatoid Arthritis
RR: Relative Risk
Th17 cells: T helper type 17 cells
UC: Ulcerative Colitis
