# Supplementary material for: The risk of rheumatoid arthritis among patients with inflammatory bowel disease: a systematic review and meta-analysis
Source: BMC Gastroenterol. 2020 Jun 17;20:192. doi: 10.1186/s12876-020-01339-3 (PMC7301504; doi:10.1186/s12876-020-01339-3)
Supplement: Supplementary file 1 — Additional file 1 Supplementary Table 1. Search strategies. [file 12876_2020_1339_MOESM1_ESM.docx]

Supplementary Table 1. Search strategies

| **PubMed** | **Embase** | **Web of Science** |
| --- | --- | --- |
| #1 "Arthritis, Rheumatoid"[Mesh]  #2 Rheumatoid Arthritis  #3 (#1 OR #2)  #4 "Inflammatory Bowel Diseases"[Mesh]  #5 Inflammatory Bowel Diseases  #6 Inflammatory Bowel Disease  #7 "Crohn Disease"[Mesh]  #8 Crohn Disease  #9 Crohn's Disease  #10 Crohns Disease  #11 Crohn Enteritis  #12 Regional Enteritis  #13 Granulomatous Enteritis  #14 Granulomatous Colitis  #15 Ileocolitis  #16 Terminal Ileitis  #17 Regional Ileitides  #18 Regional Ileitis  #19 "Colitis, Ulcerative"[Mesh]  #20 Ulcerative Colitis  #21 Colitis Gravis  #22 Idiopathic Proctocolitis  #23 (#4 OR #5 OR #6 OR #7 OR #8 OR #9 OR #10 OR#11 OR #12 OR #13 OR #14 OR #15 OR #16 OR #17 OR #18 OR #19 OR#20 OR #21 OR #22)  #24 (#3 AND #23) | #1 ' rheumatoid arthritis'/exp  #2 'rheumatoid arthritis '  #3 (#1 OR #2)  #4 'inflammatory bowel disease'/exp  #5 'inflammatory bowel disease'  #6 'inflammatory bowel diseases'  #7 'crohn disease'/exp  #8 'crohn disease'  #9 'crohns disease'  #10 'crohn enteritis'  #11 'regional enteritis'  #12 'granulomatous enteritis'  #13 'granulomatous colitis'  #14 'ileocolitis'  #15 'terminal ileitis'  #16 'regional ileitides'  #17 'regional ileitis  #18 'ulcerative colitis'/exp  #19 'ulcerative colitis'  #20 'colitis gravis'  #21 'idiopathic proctocolitis'  #22 (#4 OR #5 OR #6 OR #7 OR #8 OR #9 OR #10 OR#11 OR #12 OR #13 OR #14 OR #15 OR #16 OR #17 OR #18 OR #19 OR#20 OR #21)  #23 (#3 AND #22) | #1 Rheumatoid Arthritis  #2 Inflammatory Bowel Disease  #3 Inflammatory Bowel Diseases  #4 Crohn Disease  #5 Crohn's Disease  #6 Crohns Disease  #7 Crohn Enteritis  #8 Regional Enteritis  #9 Granulomatous Enteritis  #10 Granulomatous Colitis  #11 Ileocolitis  #12 Terminal Ileitis  #13 Regional Ileitides  #14 Regional Ileitis  #15 Ulcerative Colitis  #16 Colitis Gravis  #17 Idiopathic Proctocolitis  #18 (#2 OR #3 OR #4 OR #5 OR #6 OR #7 OR #8 OR#9 OR #10 OR #11 OR #12 OR #13 OR #14 OR #15 OR #16 OR #17)  #19 (#1 AND #18) |
