# Supplementary material for: The risk of rheumatoid arthritis among patients with inflammatory bowel disease: a systematic review and meta-analysis
Source: BMC Gastroenterol. 2020 Jun 17;20:192. doi: 10.1186/s12876-020-01339-3 (PMC7301504; doi:10.1186/s12876-020-01339-3)
Supplement: Supplementary file 2 — Additional file 2 Supplementary Table 2. Quality assessment the of included studies. [file 12876_2020_1339_MOESM2_ESM.docx]

Supplementary Table 2. Quality assessment the of included studies

| **Study** | **Design** | **Selection** | | | | **Comparability** | **Exposure** | | | **Total**  **score** |
| --- | --- | --- | --- | --- | --- | --- | --- | --- | --- | --- |
|  |  | **Case definition adequate?** | **Cases representative?** | **Selection of controls** | **Definition of controls** | **Based on design or analysis** | **Ascertainment of exposure** | **Same method for case and control** | **Non-response rate** |  |
| Puolakka-2014 | Case-control | ☆ |  | ☆ | ☆ | ☆ | ☆ | ☆ | ☆ | 7 |
| Vanessa-2019 | Case-control | ☆ |  | ☆ | ☆ | ☆☆ |  | ☆ |  | 6 |

| **Study** | **Design** | **Selection** | | | | **Comparability** | **Exposure** | | | **Total**  **score** |
| --- | --- | --- | --- | --- | --- | --- | --- | --- | --- | --- |
|  |  | **Exposed cohort representative?** | **Selection of non-exposed cohort?** | **Ascertainment of exposure** | **Outcome not presented at start?** | **Based on design or analysis** | **Assessment of outcome** | **Timing of follow-up** | **Adequate follow-up** |  |
| Aletaha-2019 | Cohort |  | ☆ | ☆ | ☆ | ☆ | ☆ | ☆ | ☆ | 7 |
| Burisch-2019 | Cohort |  | ☆ | ☆ | ☆ | ☆ | ☆ | ☆ | ☆ | 8 |
| Park-2019 | Cohort | ☆ | ☆ | ☆ | ☆ | ☆ | ☆ | ☆ | ☆ | 8 |

| **Study** | **Design** | **Selection** | | | | **Comparability** | **Exposure** | | **Total**  **score** |
| --- | --- | --- | --- | --- | --- | --- | --- | --- | --- |
|  |  | **Representative sample?** | **Sample size adequate?** | **Non-respondents** | **Ascertainment of the exposure** | **Based on design or analysis** | **Assessment of outcome** | **Statistical test** |  |
| Bae-2018 | Cross-sectional | ☆ | ☆ | ☆ | ☆ | ☆ | ☆☆ | ☆ | 7 |
| Cohen-2008-1 | Cross-sectional |  | ☆ | ☆ | ☆ | ☆ | ☆☆ | ☆ | 7 |
| Cohen-2008-2 | Cross-sectional |  | ☆ | ☆ | ☆ | ☆ | ☆☆ | ☆ | 7 |
| Hangling-2017 | Cross-sectional | ☆ | ☆ | ☆ | ☆ | ☆ | ☆☆ | ☆ | 8 |
| Kappelman-  2011 | Cross-sectional |  | ☆ | ☆ | ☆ | ☆ | ☆☆ | ☆ | 8 |
| Weng-2007 | Cross-sectional | ☆ | ☆ | ☆ | ☆ | ☆☆ | ☆☆ | ☆ | 9 |
| Yang-2018 | Cross-sectional |  | ☆ | ☆ | ☆ |  | ☆☆ | ☆ | 6 |
