# Supplementary material for: The risk of rheumatoid arthritis among patients with inflammatory bowel disease: a systematic review and meta-analysis
Source: BMC Gastroenterol. 2020 Jun 17;20:192. doi: 10.1186/s12876-020-01339-3 (PMC7301504; doi:10.1186/s12876-020-01339-3)
Supplement: Supplementary file 3 — Additional file 3 Supplementary Figure 1. Publication bias assessed by Begg’s test and Egger’s test. [file 12876_2020_1339_MOESM3_ESM.docx]

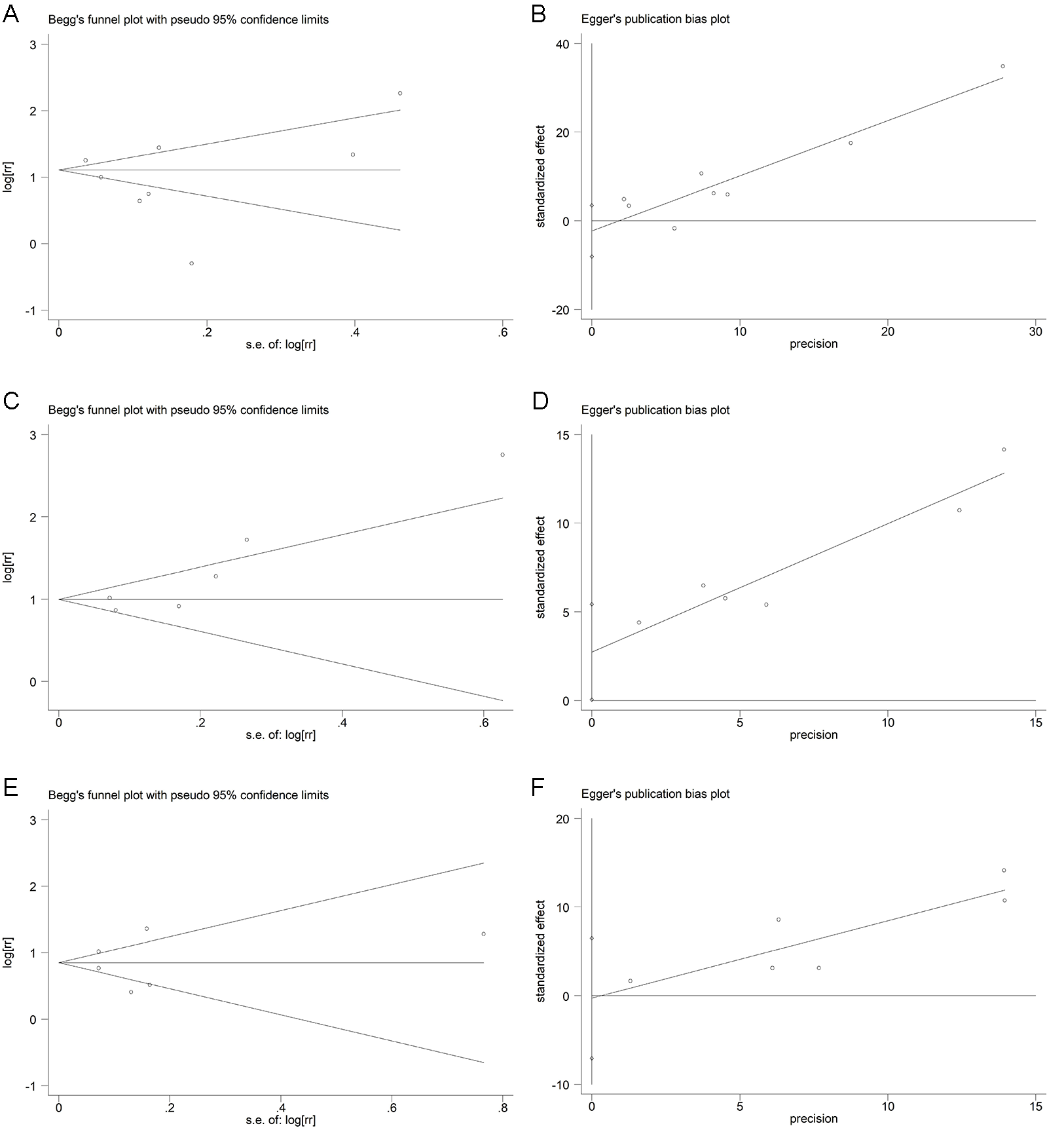


Supplementary Fig. 1 Publication bias assessed by Begg’s test and Egger’s test. The risk of rheumatoid arthritis among patients with inflammatory bowel disease: A, Begg’s test (P=0.902); B, Eggers’ test (P=0.369). The risk of rheumatoid arthritis among patients with Crohn disease: C, Begg’s test (P=0.060); D, Eggers’ test (P=0.048). The risk of rheumatoid arthritis among patients with ulcerative colitis: E, Begg’s test (P=1.000); F, Egger’s test(P=0.914).
