# Supplementary material for: The risk of rheumatoid arthritis among patients with inflammatory bowel disease: a systematic review and meta-analysis
Source: BMC Gastroenterol. 2020 Jun 17;20:192. doi: 10.1186/s12876-020-01339-3 (PMC7301504; doi:10.1186/s12876-020-01339-3)
Supplement: Supplementary file 4 — Additional file 4 Supplementary Table 3. Sensitivity analysis. [file 12876_2020_1339_MOESM4_ESM.docx]

Supplementary Table 3. Sensitivity analysis

| **Sensitivity**  **analysis** | **Removed study** | **Summary based on remained studies (random-effects model)** | | |
| --- | --- | --- | --- | --- |
|  |  | **Relative risk (95% CI)** | **P value** | **Heterogeneity (I^2^)** |
| RA in IBD | None | 2.59 (1.93-3.48) | 0.000 | 94.2% |
|  | Aletaha-2019 | 2.50 (1.72-3.64) | 0.000 | 92.6% |
|  | Burisch-2019 | 2.69 (1.94-3.71) | 0.000 | 94.7% |
|  | Cohen-2008 | 2.64 (1.75-4.00) | 0.000 | 94.9% |
|  | Kappelman-2011 | 2.37 (1.76-3.20) | 0.000 | 94.8% |
|  | Park-2019 | 2.40 (1.74-3.31) | 0.000 | 94.8% |
|  | Puolakka-2014 | 3.01 (2.38-3.79) | 0.000 | 89.7% |
|  | Vanessa-2019 | 2.51 (1.85-3.42) | 0.000 | 95.0% |
|  | Weng-2007 | 2.73 (1.99-3.74) | 0.000 | 94.1% |
| RA in CD | None | 3.14 (2.46-4.01) | 0.000 | 74.9% |
|  | Burisch-2019 | 3.09 (2.35-4.05) | 0.000 | 78.1% |
|  | Cohen-2008-1 | 3.58 (2.41-5.31) | 0.000 | 79.8% |
|  | Cohen-2008-2 | 3.62 (2.56-5.10) | 0.000 | 74.2% |
|  | Kappelman-2011 | 2.89 (2.37-3.54) | 0.000 | 66.8% |
|  | Park-2019 | 2.82 (2.27-3.50) | 0.000 | 67.4% |
|  | Weng-2007 | 3.39 (2.52-4.55) | 0.000 | 79.7% |
| RA in UC | None | 2.29 (1.76-2.97) | 0.000 | 84.9% |
|  | Burisch-2019 | 2.44 (1.83-3.25) | 0.000 | 86.0% |
|  | Cohen-2008-1 | 2.18 (1.56-3.05) | 0.000 | 83.7% |
|  | Cohen-2008-2 | 2.34 (1.60-3.40) | 0.000 | 87.2% |
|  | Kappelman-2011 | 2.26 (1.73-2.95) | 0.000 | 87.8% |
|  | Park-2019 | 2.05 (1.59-2.63) | 0.000 | 81.6% |
|  | Weng-2007 | 2.52 (1.96-3.24) | 0.000 | 80.2% |

CD, Crohn Disease; IBD, Inflammatory Bowel Disease; RA, Rheumatoid Arthritis; UC, Ulcerative Colitis.
